# Tiny but significant: on the importance of thrips as pollinators

**DOI:** 10.1093/aob/mcaf069

**Published:** 2025-04-17

**Authors:** Cristina Pop, Irene Terry, Laurence A Mound, Casper J van der Kooi

**Affiliations:** Groningen Institute for Evolutionary Life Sciences, University of Groningen, Groningen NL-9747AG, The Netherlands; School of Biological Sciences, University of Utah, Salt Lake City, UT 84112 USA; Australian National Insect Collection CSIRO, Canberra, ACT 2601, Australia; Groningen Institute for Evolutionary Life Sciences, University of Groningen, Groningen NL-9747AG, The Netherlands

**Keywords:** Thysanoptera, pollination, mutualism, evolution, floral trait, pollen load, meta-analysis, cycads, floral architecture, pollination mutualism

## Abstract

**Background:**

Thrips (Thysanoptera) are minute plant and flower visitors in ecosystems across the world but are commonly viewed as notorious pests and too small to effect pollination. The role of thrips as pollinators is thus largely neglected. We provide an overview of the number of plant taxa that are pollinated by thrips, the floral traits of thrips-pollinated plants, and discuss why thrips can be effective pollinators.

**Main findings:**

Thrips pollination occurs in almost half of all seed plant orders, 53 families and 102 plant genera. In many taxa, thrips are the primary or only pollinator. Thrips effectiveness as pollen vectors is enhanced by enormous thrips population sizes in inflorescences, pollen loads of up to >100 grains per individual, and the ability to travel in wind streams. A meta-analysis shows that thrips can significantly contribute to seed and fruit set compared with open pollination controls. A review of the floral traits of thrips-pollinated plants suggests that there is no universal ‘thripophily’ pollination syndrome. One plant trait that stands out is a floral architecture that limits access to larger pollinators and gives thrips a refuge, such as globose or disc-like structures with small or slit-like openings. Some specialist systems exhibit characteristic floral or cone odours that attract thrips; however, too few systems have been studied in detail to cast a general description. Together, our results suggest that pollination by thrips is more common than has been historically perceived, and thrips should not be overlooked in pollination studies and as agents of selection of floral traits.

## INTRODUCTION

Nearly 90 % of the world’s flowering plant species depend on animals for their reproduction (Ollerton *et al.*, 2011). Scholarly and media attention on pollinating animals typically focuses on the most charismatic species, such as bees, birds and butterflies, but these species are not necessarily representative of the broad diversity of animals that do pollinate flowers. Numerous often less aesthetic and commonly unloved animals, such as earwigs, cockroaches, tabanids, fungus gnats and isopods, can act as pollinators (Wardhaugh, 2015; Ollerton, 2017; Mochizuki *et al.*, 2023; Johnson, 2024; Suetsugu, 2025). Another group for which the role of pollinator is generally overlooked is thrips (order Thysanoptera). Thrips (both singular and plural) are minute insects (typically 1 mm) with a notorious reputation as crop pests, which has led to their role as pollinators having been largely ignored (Mound, 2004). We bring attention to thrips as pollinators by examining the results of more than a century of literature on thrips pollination.

The role of thrips as pollinators is relevant for at least four reasons. First, thrips are hailed as some of the primeval pollinators in the early history of gymnosperms and flowering plants (Labandeira, 1998; Labandeira *et al.*, 2007; Peñalver *et al.*, 2012; Ollerton, 2017; Peña-Kairath *et al.*, 2023, 2024; Guo *et al.*, 2024; Peñalver *et al.*, 2025). Thrips have been found associated with cycadopite pollen from early Cretaceous amber deposits. The earliest definitive age of thrips is from Triassic compression fossil formations in Virginia (eastern USA) and Kazakhstan (Grimaldi *et al.*, 2004), and to date there are ~7000 species described. Thus, the fossil record hints at an important role for thrips as pollinators of gymnosperms. An example of an extant thrips–gymnosperm pollination system is the cycads (Cycadales), which were an important terrestrial dioecious plant group in the Mesozoic (Norstog and Nicholls, 1997). Many extant cycads in the genus *Macrozamia* (Zamiaceae) are exclusively pollinated by a specialist thrips from a basal thrips family (Forster *et al.*, 1994; Toon *et al.*, 2020; Mound and Terry, 2001). Uncovering how the plant and pollinator interact may identify traits fundamental to the functioning of early pollination systems (van der Kooi and Ollerton, 2020). Second, previous general literature on pollination has frequently left unnoticed the associations of flowers and thrips, as thrips are mostly assumed to be pestiferous herbivores or disease vectors and are not able to transport enough pollen to be effective. In these cases, pollination may have been incorrectly assigned to wind or bees, even when bees did not actually visit these flowers (also see the discussion in Scott-Brown *et al.*, 2019). Excluding thrips in studies on pollination and plant reproductive success thus yields an incomplete or even erroneous picture of plant reproduction. Third, although historically thrips have largely been ignored as pollinators, there is a growing body of evidence revealing that thrips play a prominent role in ecosystems as pollinators of key plant species (Sakai, 2002; Scott-Brown *et al.*, 2019; Henderson, 2024), meaning they are significant for conservation and habitat management. Fourth, thrips are implicated in contributing to the pollination of many crop species (Kirk, 1997; Lewis, 1997). Thus, in view of food production and security, thrips pollination should be considered.

In this paper, we highlight the significance of thrips as pollinators of wild and crop plants through several complementary approaches. We first present a short background on the insect order Thysanoptera and discuss which thrips families have flower-visiting species. We then provide an overview of the extent of plant orders in which pollination by thrips is documented. We distinguish between correlative and experimental evidence on the role of thrips pollinators, followed by a discussion on what aspects of the life history of thrips render them effective as pollinators. Through a meta-analysis we show that in studies where the importance of thrips was quantitatively investigated thrips were found to contribute significantly to reproductive fitness. Finally, we discuss typical floral traits of plants (‘thripophily’) that are largely pollinated by thrips, including taxa where thrips are the sole pollinator.

## BIOLOGICAL DIVERSITY AMONG THRIPS TAXA AND THEIR LINK TO FLOWERS

To understand the role of thrips as pollinators, we first briefly introduce some key elements about the insect order Thysanoptera, which comprises two suborders, Terebrantia and Tubulifera. We consider aspects of the basic systematics, the diversity of biologies exhibited, and what deductions might be made about the origin of their association with flowers and possible significance as pollinators. In this, it is important to consider how few Thysanoptera species are flower-living as well as that most thrips are feeding on fungi or leaves. Of the 15 Thysanoptera families, six are only known from fossils described as members of Terebrantia. The Tubulifera comprises only a single, albeit speciose, family, the Phlaeothripidae. About 60 % of the 4000 Phlaeothripidae are fungus-feeding, living on dead branches and dead leaves. Of the remaining 40 % of Phlaeothripidae species, the majority are leaf-feeding with many also gall-inducing. Only a few species of Phlaeothripidae are flower-living, and these are mainly members of the species-rich genus *Haplothrips*. Within the extant species of the Terebrantia suborder, there are ~3000 species in eight families. The Fauriellidae comprises just five species of little-known biology (Mound and Hastenpflug-Vesmanis, 2021). Two small families, Merothripidae and Uzelothripidae, comprise <20 species, all of which are presumably fungus-feeding. The insects that are commonly considered ‘flower thrips’ are among the 2200 species of the Thripidae, which is the second largest Thysanoptera family, although many Thripidae species are obligate leaf-feeders. The remaining four families, Aeolothripidae, Heterothripidae, Melanthripidae and Stenurothripidae, comprise nearly 400 species, and almost all of these are flower-living. Melanthripidae and Stenurothripidae are known to have associations with early Cretaceous cycadopite pollen (Peñalver *et al.*, 2012; Peña-Kairath *et al.*, 2023; Guo *et al.*, 2024). Based on the variation of feeding habits within and across families as described above in combination with phylogenetic analyses, floral feeding has likely arisen several times. For example, molecular phylogenetic analyses of Thysanoptera (Buckman *et al.*, 2013) suggest that the ancestral feeding type was fungal and leaf feeding. Morphological phylogenetic analyses of the family Thripidae (Zhang *et al.*, 2019) suggests that leaf feeding was the ancestral state from which floral feeding was derived, and subsequently there have been several reversals between leaf and floral feeding.

Thrips in flowers can commonly be observed bearing pollen grains on their bodies even when not experimentally studied. Such observations may be discounted as pollen feeding, implying that this prohibits the possibility that they also transfer pollen. As an example, the relatively large and often bicoloured thrips of *Aeolothrips*, *Desmothrips* and *Melanthrips* (all Aeolothripidae) breed only in flowers, and move from flower to flower, usually bearing pollen on their bodies. Another group for which thrips pollination is often not considered is Fabaceae flowers. Although thrips cannot trip open keel petals in the way that larger insects (e.g. bumblebees) can, they can squeeze their way in and out. Many thrips species around the world live only in Fabaceae flowers and transport their pollen (Mound *et al.*, 2018; Mound and Tree, 2020). Thrips of the genera *Odontothrips* in the Palaearctic and *Odontothripiella* in Australia breed only in Fabaceae flowers, which they enter through small openings at the keel or around the stem. The host associations of many of these species often involve species specificity (Mound *et al.*, 2018; Mound and Tree, 2020). Fabaceae flowers are commonly considered to have co-evolved with bees as pollinators, which might lead to overlooking thrips as alternative pollen vectors.

Strict host specificity is relatively unusual among flower thrips (Mound and Tree, 2020), but there are two early-radiating genera within two families that have strong host associations. Among the 23 genera of Aeolothripidae, *Cycadothrips* species breed only in the pollen cones of some *Macrozamia* cycad species, and that biology is discussed later. The family Stenurothripidae comprises ten genera of fossil thrips, of which fossils have been found in association with cycadopite pollen (Peña-Kairath *et al.*, 2023, 2024; Guo *et al.*, 2024) and another with undescribed pollen (Ulitzka, 2024), and three genera of living thrips. In the genus *Holarthrothrips*, found between India and the Mediterranean area, three species live only in the male flowers of *Phoenix*, including *P. dactylifera*, the date palm (Bhatti, 1986; Mound and Tree, 2020). Little is known of pollinators of *P. dactylifera* in its native regions (Henderson, 2024), so it is possible that these thrips are the natural pollinators of this valuable tree crop now grown throughout warm climates.

## THRIPS ARE POLLINATORS ACROSS GYMNOSPERMS AND ANGIOSPERMS

To obtain an overview of the number of plant taxa with thrips pollination, we collated all scientific observations of thrips as putative pollinators. Given the variation in the degree of certainty as to whether thrips are pollinators or mere flower visitors, we classified observations from literature into three groups. (a) Thrips pollination is experimentally demonstrated and the effect size quantified. This category includes all papers selected for the meta-analysis (see below), and some additional studies with slightly different experimental designs that demonstrate thrips pollination quantitatively. (b) Thrips pollination is very likely, but the biological effect size is not quantified. Case studies in this category were mainly based on observations of thrips carrying pollen, often in conjunction with observations of between-flower movement of thrips. (c) Thrips pollination is suggested but unquantified. These case studies were based on observations of large numbers of thrips inside the flower/inflorescence.

**Fig. 1.**
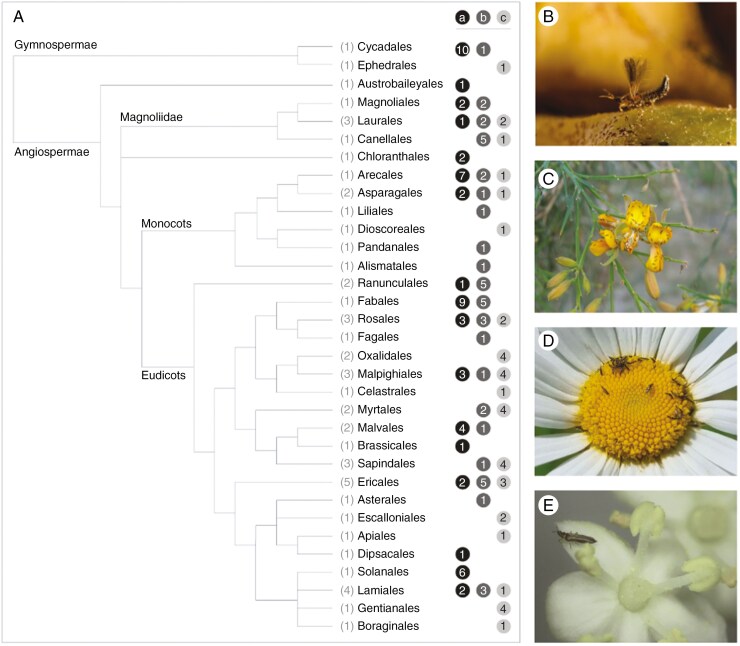
Plant orders in which thrips pollination is known or likely to occur. (A) Phylogeny with taxa for which thrips pollination is reported. The number of families with thrips pollination per order is given in parentheses. Grey-scale coding on the right indicates the type of evidence suggesting thrips pollination with the number of species per category: (a) experimentally validated; (b) strong indirect evidence; (c) only indirect evidence (see main text). Phylogeny after the Angiosperm Phylogeny Group (Christenhusz *et al.*, 2011; Chase *et al.*, 2016). (B) A *Cycadothrips chadwicki* female exiting a *Macrozamia* pollen cone (Cycadales; credit James Dorey). (C) *Odontothripiella* sp. (black dots) swarming on a *Jacksonia* flower (credit Glynn Maynard). (D) thrips on *Leucanthemum* cf. *vulgaris* (Asterales; credit Sara Leonhardt). (E) A thrips on *Sambucus nigra* (Dipsacales; credit Alison Scott-Brown).

Pollination by thrips occurs across Angiosperms and Gymnosperms widely and there is no indication of phylogenetic associations (Fig. 1). In total, we found evidence suggesting thrips pollination in 33 seed plant orders (almost half of all seed plant orders), 53 plant families and 139 plant species (Fig. 1; Supplementary Data). These numbers certainly underrepresent the real scope of thrips pollination, given thrips are understudied and considered a nuisance or pest rather than a pollinator.

Thrips pollination is well studied in several plant orders. The best studied order with respect to thrips pollination is Cycadales, which comprises at least 12 species for which thrips pollination was experimentally validated or observed. Though most cycads across the world are pollinated by beetles, one exception is within the genus *Macrozamia*, endemic to Australia, where thrips in the genus *Cycadothrips* are either the sole or co-pollinator of many species (reviewed by Toon *et al.*, 2020). Fabales, Arecales and Solanales encompass several well-studied thrips-pollinated groups. In the Fabales we have some of the earliest reports of thrips pollination (Annand, 1926), followed by later studies (Velayudhan *et al.*, 1985; Annadurai and Velayudhan, 1986). Within the Arecales, of the 149 species of palm that have been studied, only 5 % are thrips-pollinated (Henderson, 2024). Two palm species are pollinated by polyphagous thrips: *Elaeis guineënsis*, the oil palm, grown in non-native areas in peninsular Malaysia, is pollinated by *Thrips hawaiiensis* (Syed, 1979), and *Linospadix monostachyus* in Australia is associated with *Thrips setipennis* (Williams *et al.*, 2001). The rest of the thrips-pollinated palms have specialist thrips. *Brooksithrips chamaedorea* pollinates many species of the dioecious Neotropical palm *Chamaedorea* (Ríos *et al.*, 2014; Morgan, 2007) and *Holarthrothrips* sp. is likely a pollinator of the Old-World date palm, *Phoenix dactylifera* (Mound and Tree, 2020). Another reasonably well-studied order is Ericales. *Ceratothrips ericae* is noted as a common visitor/pollinator of shrubby Ericales including *Arctostaphylos uva-ursi*, *A. pungens* and *Calluna vulgaris* (Hagerup, 1950; García-Fayos and Goldarazena, 2008; Eliyahu *et al.*, 2015). Other species within *Vaccinium, Napoleonaea*, *Cyclamen*, *Diospyros* and *Rapanea* are associated with different thrips species as demonstrated or putative pollinators (Frame and Durou, 2001; Williams *et al.*, 2001; Schwartz-Tzachor *et al.*, 2006; Gaines-Day and Gratton, 2015; Sircom, 2017).

Pollination in the mulberry family (Moraceae) offers some intriguing connections between two subfamilies, the Castilleae and Ficeae (*Ficus*). For one, thrips pollinators of Castilleae are found in both Palaeotropics and Neotropics, *Thrips antiaropsidis* specializes only on *Antiaropsis decipiens* in New Guinea and *Frankliniella diversa* on *Antiaropsis decipiens* in Panama (Sakai, 2001; Zerega *et al.*, 2004). Three other genera of this subfamily, two Neotropical and one Palaeotropical, have reported thrips as pollinators. The pollination systems of the remaining genera are unknown. Second, Castilleae is the sister taxon of Ficeae (Datwyler and Weiblen, 2004)*. Ficus* species are pantropical and pollinated by obligate specialist agaonid wasps. Consistent with this taxonomic connection is the floral morphological adaption whereby the involucral bracts enclose the flowers, resulting in entomophily in these two subfamilies with anemophilous ancestry in which flowers have no enclosing bracts (Datwyler and Weiblen, 2004; Moe *et al.*, 2012). The *Ficus*–fig wasp pollination system has been a rich resource for the study of the evolution of specialist pollination systems and species radiation. Given the many unknowns about thrips pollination and the lack of pollinator study of many Castilleae genera, thrips pollination may be more widespread, and Castilleae offers potential for understanding trait evolution leading to pollinator specialization in these subfamilies.

## LIFE HISTORY TRAITS OF THRIPS THAT UNDERSCORE THEIR ROLE AS POLLINATORS

**Fig. 2.**
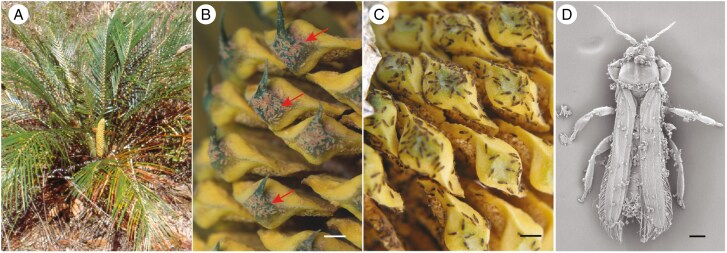
*Cycadothrips chadwicki* on *Macrozamia* pollen cone during thermogenesis. (A) Habitus picture of *M. mountperriensis*. (B, C) Larvae (B, arrows) and adults (C) exiting an *M. macleayi* pollen cone (Terry *et al.*, 2014). (D) Adult thrips covered in pollen; captured immediately after walking to the edge of a sporophyll before grooming and taking flight (credit: Desley Tree). Scale bars: (B, C) = 1 cm; (D) = 100 μm.

Several life history traits of thrips underline their potential as pollinators. First, the sheer number of individual thrips that can occur at a single inflorescence warrants an evaluation of their capacity to disperse pollen (Figs 1 and 2). An extreme example of the sheer population size of thrips on plant reproductive organs is presented by Mound and Terry (2001), who caught >4000 individual thrips arriving on a single *Macrozamia* ovulate cone in 2 hours. Similarly, in a single staminate *Chamaedorea* inflorescence >2000 individuals were caught (Ríos *et al.*, 2014). These extreme numbers of thrips generally occur because many thrips species use flowers or cones as brood sites, and thrips have short generation times (Sakai, 2002). The larvae and adults feed on floral tissue, nectar and pollen. Generation times of thrips can be very short, with just 1–2 weeks in some species (Sakai, 2002), meaning that thrips can produce several generations within one flowering season. Populations can grow even faster for species that reproduce asexually, which is common in several thrips families and induced by parthenogenesis-inducing endosymbionts (Lewis, 1973; van der Kooi and Schwander, 2014; van der Kooi *et al.*, 2017).

Individual pollen load can predict the pollination potential of a putative pollinator (Dafni, 1992). To obtain an overview of the potential pollen transfer by individual thrips, we collated pollen counts on individual thrips from the literature (Table 1). We searched for papers using the keywords ‘thrips’, ‘pollen count’ and ‘pollen load’ in different combinations in Google Scholar. Pollen loads were extracted from 18 papers, along with the respective thrips and plant species. We extracted pollen load data from 17 studies on 37 plant species, which revealed a pattern of considerable variation among the different plant species and their associated thrips species. The observed pollen loads ranged from as low as one grain per individual for *Brooksithrips chamaedoreae* visiting *Chamaedorea tepejilote* (Aracaceae) to as high as 175 grains per individual for *Frankliniella schultzei* visiting *Tridax procumbens* (Asteraceae). The average number of pollen grains per individual was 36. There were eight accounts of pollen loads of >100 grains per individual, observed for species of *Haplothrips*, *Frankliniella*, *Megalurothrips* and *Cycadothrips* (Table 1). The wide range of variation of pollen loads across thrips species could be due to several factors, e.g. size, density and structure of thrips setae, loss of pollen due to grooming and wind, and the amount of pollen presented in flowers or cones, as well as differences in the size and structure of pollen grains. Clearly, the pollen load of individual thrips can be relatively high and, together with the vast numbers of individual thrips on inflorescences (Figs 2C and 2D), it can render thrips effective pollinators.

**Table 1.** Thrips pollen load for different plant taxa. Pollen load is measured as pollen grains/individual.

Plant family	Plant taxon	Thrips species	Pollen load	Reproductive structure	Reference
Arecaceae	*Elaeis guineensis*	*Thrips hawaiiensis*	4.8	Pistillate flowers	Syed, 1979
	*Chamaedorea tepejilote*	*Brooksithrips chamaedoreae*	1	Pistillate flowers	Ríos *et al.*, 2014
	*C. costaricana*	*B. chamaedoreae*	1.1	Pistillate flowers	Ríos *et al.*, 2014
	*C. pinnatifrons*	*B. chamaedoreae*	2.5	Pistillate flowers	Ríos *et al.*, 2014
Asteraceae	*Wedelia chinensis*	*Frankliniella schultzei*	150	Bisexual flowers	Ananthakrishnan, 1982
	*W. chinensis*	*Megalurothrips distalis*	120	Bisexual flowers	Ananthakrishnan, 1982
	*W. chinensis*	*Micothrips fasciatus*	42.5	Bisexual flowers	Ananthakrishnan, 1982
	*W. chinensis*	*Microcephalothrips abdominalis*	47.5	Bisexual flowers	Ananthakrishnan, 1982
	*W. chinensis*	*Haplothrips gowdeyi*	105	Bisexual flowers	Ananthakrishnan, 1982
	*W. chinensis*	*Haplothrips tardus*	115	Bisexual flowers	Ananthakrishnan, 1982
	*Tridax procumbens*	*F. schultzei*	175	Bisexual flowers	Ananthakrishnan, 1982
	*T. procumbens*	*M. abdominalis*	57.5	Bisexual flowers	Ananthakrishnan, 1982
	*T. procumbens*	*H. gowdeyi*	152.5	Bisexual flowers	Ananthakrishnan, 1982
	*T. procumbens*	*H. tardus*	140	Bisexual flowers	Ananthakrishnan, 1982
	*Cosmos bipinnatus*	*M. abdominalis*	95	Bisexual flowers	Ananthakrishnan, 1982
	*C. bipinnatus*	*T. hawaiiensis*	75	Bisexual flowers	Ananthakrishnan, 1982
	*Ageratum conyzoides*	*M. abdominalis*	70	Bisexual flowers	Ananthakrishnan, 1982
	*Vernonia cinerea*	*H. gowdeyi*	52.5	Bisexual flowers	Ananthakrishnan, 1982
	*Bellis perennis*	*Frankliniella minuta*	2	Bisexual flowers	Annand, 1926
Brassicaceae	*Arabidopsis thaliana* var. *Columbia*	*Frankliniella occidentalis*	4.6	Bisexual flowers	Booij *et al.*, 2016
Dipterocarpaceae	*Shorea acuminata*	*Thrips* spp.	8.1	Bisexual flowers	Kondo *et al.*, 2016
Euphorbiaceae	*Macaranga hullettii*	*Neoheegeria* sp.	37	Pistillate flowers	Moog *et al.*, 2002
	*M. hullettii*	*Neoheegeria* sp.	63.6	Staminate flowers	Moog *et al.*, 2002
Ericaceae	*Arctostaphylos pungens*	*Orothrips kelloggii*, *Oligothrips oreios*	4.6	Bisexual flowers	Eliyahu *et al.*, 2015
Fabaceae	*Vigna catjang*	*F. schultzei*	9	Bisexual flowers	Annadurai and Velayudhan, 1986
	*Medicago sativa*	*F. tritici*	2.3	Bisexual flowers	Annand, 1926
	*Acacia* sp.	*F. tritici*	1	Bisexual flowers	Annand, 1926
	*Lupinus* sp.	*F. tritici*	26.5	Bisexual flowers	Annand, 1926
Moraceae	*Castilla elastica*	2 *Frankliniella* spp.	7.6	Complementary inflorescences	Sakai, 2001
Moraceae	*C. elastica*	2 *Frankliniella* spp.	25.8	Staminate inflorescences	Sakai, 2001
Oleaceae	*Syringa* sp.	*F. tritici*	14	Bisexual flowers	Annand, 1926
	*Syringa* sp.	*Thrips madronii*	6	Bisexual flowers	Annand, 1926
	*Syringa* sp.	*Aeolothrips kuwanaii*	13	Bisexual flowers	Annand, 1926
Onagraceae	*Clarkia unguiculata*	*F. tritici*	1.5	Bisexual flowers	Annand, 1926
Papaveraceae	*Eschscholzia californica*	*F. tritici*	3.3	Bisexual flowers	Annand, 1926
	*E. californica*	*F. minuta*	2	Bisexual flowers	Annand, 1926
	*E. californica*	*Sericothrips apteris*	3	Bisexual flowers	Annand, 1926
Rosaceae	*Prunus* sp.	*F. tritici*	0.3	Bisexual flowers	Annand, 1926
	*Prunus* sp.	*Taeniothrips inconsequens*	1.3	Bisexual flowers	Annand, 1926
	*Prunus laurocerasus*	*T. inconsequens*	19	Bisexual flowers	Annand, 1926
	*P. laurocerasus*	*F. tritici*	4	Bisexual flowers	Annand, 1926
Rubiaceae	*Coffea arabica*	7 *Frankliniella* spp.	1.7	Bisexual flowers	Infante *et al.*, 2017
	*Coffea canephora*	7 *Frankliniella* spp.	1.7	Bisexual flowers	Infante *et al.*, 2017
Schisandraceae	*Schisandra sphenanthera*	*Thrips flavidulus*	14.54	Staminate flowers	Du *et al.*, 2012
Solanaceae	*Solanum melongena*	*F. schultzei*	28	Bisexual flowers	Velayudhan and Annadurai, 1986
	*Capsicum frutescens*	*F. schultzei*	19	Bisexual flowers	Velayudhan and Annadurai, 1986
	*Solanum torvum*	*F. schultzei*	16	Bisexual flowers	Velayudhan and Annadurai, 1986
	*S. xanthocarpum*	*F. schultzei*	18	Bisexual flowers	Velayudhan and Annadurai, 1986
	*S. trilobatum*	*F. schultzei*	21	Bisexual flowers	Velayudhan and Annadurai, 1986
	*S. nigrum*	*F. schultzei*	28	Bisexual flowers	Velayudhan and Annadurai, 1986
Zamiaceae	*Macrozamia communis*	*Cycadothrips chadwicki*	52.1	Pollen cones	Terry, 2001
	*M. communis*	*C. chadwicki*	21.6	Ovulate cones	Terry, 2001
	*Macrozamia macdonnellii*	*C. albrechti*	20.5	Pollen cones	Mound and Terry, 2001
	*M. macdonnellii*	*C. albrechti*	15.1	Ovulate cones	Mound and Terry, 2001
	*Macrozamia lucida*	*C. chadwicki*	107	Pollen cones	Terry *et al.*, 2005
	*M. lucida*	*C. chadwicki*	29.2	Ovulate cones	Terry *et al.*, 2005
Winteraceae	*Belliolum pauciflorum*	*Taeniothrips novocaledonensis*	1.2	Bisexual flowers	Thien *et al.*, 2000

In addition to the number of individuals and their pollen-carrying capacity, pollinator travel distance is an important element for both plant outcrossing success and population genetics. Thrips are not the best fliers—their flying behaviour is sometimes referred to as ‘passive parachuting’ (Santhanakrishnan *et al.*, 2014)—but this does not mean that they cannot travel far. High-speed video recording and wing kinematic modelling suggested that bristled wings aid thrips dispersal (Santhanakrishnan *et al.*, 2014). Most literature on thrips dispersal focuses on human-mediated introduction of thrips to new environments (Mound, 2004; Morse and Hoddle, 2006); however, there is evidence of long-range natural dispersal. Given their high surface-to-volume ratio, thrips can be transported by air currents and are sometimes called ‘aerial plankton’ (*sensu*  Mound, 1983). Experiments using aerial trapping of flying insects resulted in grass thrips being caught at altitudes >300 m in the air (Lewis, 1965). Mound (1983) found several examples of New Zealand thrips fauna that have close connections with Australian thrips, including the wingless *Anaphothrips*, which might be owing to natural dispersal from Australia via aerial wind transport along the Tasman Sea. As part of a larger study on the distribution of insects in the air, Glick (1939) caught thrips at altitudes >3000 m. How these long-range travel events affect pollen load transport has not been studied. Together, these examples nevertheless attest to the huge dispersal potential of these tiny insects.

In a few field studies, researchers have used observations of plant infestation or capture of thrips relative to the distance from putative source populations to estimate dispersal ability of thrips. In one example, the distribution patterns of the gall-inducing thrips *Oncothrips tepperi* in the field were used to estimate dispersal distances of >120 m (McLeish *et al.*, 2003), though this would benefit from further investigation. In a greenhouse experiment, *Frankliniella occidentalis* individuals were released, and an average dispersal rate of 0.05–0.17 m d^−1^ was found (Rhainds and Shipp, 2004). Investigating *Cycadothrips albrechti* movement between cones of *Macrozamia macdonnellii* in canyons of central Australia, Mound and Terry (2001) observed that *Cycadothrips* leaving pollen cones consistently moved upward towards the sky. On one day, they observed that the only receptive ovulate cone attracting thrips was one canyon from the nearest pollen cone, estimated at 60 m away. This suggests that there can be directed movement from a cone source towards attractive cones after aerial dispersal.

For self-pollinated plants, thrips dispersal ability is less important. Baker and Cruden (1991) indicated thrips-mediated selfing in *Potentilla rivalis* (Rosaceae) and *Ranunculus sceleratus* (Ranunculaceae), though outcrossing in these species was attributed to bees. A similar scenario was suggested for *Habenaria radiata* (Orchidaceae), which is primarily adapted to and pollinated by hawkmoths, but thrips contribute significantly to seed set, presumably through thrips-mediated selfing (Shigeta and Suetsugu, 2020). Thrips also facilitate self-pollination in the tree *Shorea acuminata* (Dipterocarpaceae), though genetic analyses revealed that *Geocoris*, a bug that preys upon thrips, contributes most to outcrossing (Kondo *et al.*, 2011). A combination of thrips-mediated selfing and outcrossing by other insects probably occurs in more systems (e.g. Solomon Raju *et al.*, 2022). In plants for which thrips are not the main pollinator, fast population size/growth of thrips may contribute to long-term reliability when the main pollinator is absent. In other words, the presence of thrips may facilitate autogamy or geitonogamy and thus confer reproductive assurance for self-compatible plants when other insects are scarce. The exact fitness benefit of any of such thrips-pollinated selfing in presence/absence of other (outcrossing) insects currently is unknown.

## QUANTITATIVE ANALYSIS OF THE CONTRIBUTION OF THRIPS TO PLANT REPRODUCTIVE SUCCESS

To quantify the importance of thrips for reproduction of plants we carried out a meta-analysis. First, we systematically searched Google Scholar using a combination of specific keywords: ‘thrips’, ‘Thysanoptera’, ‘pollination’, ‘pollinator’ and ‘exclusion’. Second, for all papers that appeared potentially relevant, we carefully examined the experimental design, the statistics applied, and whether the study reported all necessary parameters. We incorporated studies that used fruit set, seed set or both as measures of reproductive success. All articles selected for our analysis included three types of pollination treatments in their experimental design: (1) open pollination, where access of pollinators was unmanipulated; (2) exclusion of all insects except thrips (thrips only); (3) exclusion of all pollinators to test for the impact of autonomous self-pollination and other modes of uniparental reproduction. The selective exclusion of insects is typically done by covering inflorescences with a net of different mesh sizes. For example, a 1-mm-mesh net was used to allow access to only thrips and a paper bag or a finer mesh net (0.2 mm) was used to exclude all insects from the inflorescence. Third, we cross-checked references in and to all papers included in our dataset to find additional studies that had not surfaced in the initial search. The literature search was done in December 2023. In total, we found 12 papers that fitted all criteria, yielding data for 13 plant species. One study included two species, *Potentilla rivalis* and *Ranunculus sceleratus* (Baker and Cruden, 1991), and for one species, *Arctostaphylos uva-ursi*, two separate populations were studied (García-Fayos and Goldarazena, 2008).

As an effect size, we used the log response ratio (also called log-transformed ratio of means) (Hedges *et al.*, 1999), which was computed using the metafor package in R (Viechtbauer, 2010). We calculated the effect sizes of open pollination relative to thrips-only. Seed/fruit set values for inflorescences for which all insects were excluded (treatment type 3, above) were subtracted from values for open and thrips-only treatment to correct for uniparental reproduction. A random effects model (restricted maximum likelihood), implemented through the ‘rma’ function, was used to calculate a combined effect size across all studies.

**Fig. 3.**
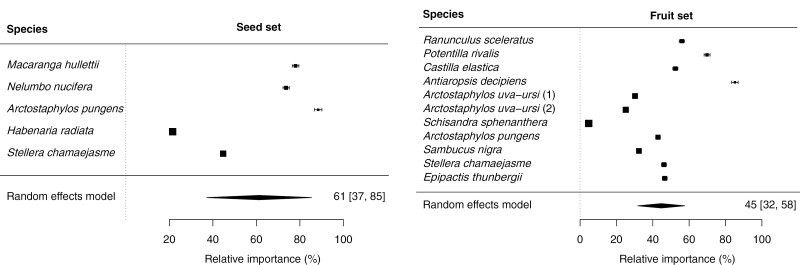
Relative importance ( %) of thrips for seed set (left) and fruit set (right) compared with open pollination and corrected for uniparental reproduction for each plant species. The mean per category is shown at the bottom. Data are mean effects ± 95  % confidence intervals; square size is proportional to sample size. Percentages and confidence intervals are back-transformed values of the log response ratio values. The percentages represent how close the thrips-only treatment is to the open pollination treatment.

Our meta-analysis shows that the reproductive output measured through seed set or fruit set is considerable owing to pollen transfer by thrips. The average contribution of thrips to seed and fruit set in these plants is 61 and 45 %, respectively, relative to open pollination (Fig. 3). The mean responses are significantly different from open pollination (seed set: d.f. = 4, *z* = 2.3, *P* = 0.02; fruit set: d.f. = 10, *z* = 4.7, *P* < 0.001), which means that, overall, reproductive output is reduced when all non-thrips insects are barred from the reproductive organs. However, the contribution of thrips alone to these plants’ reproductive success can be (very) high. The highest values are found for *Arctostaphylos pungens*, for which 88 % of seed set is from thrips (Eliyahu *et al.*, 2015), and *Antiaropsis decipiens*, for which fruit set is 85 % from thrips (Zerega *et al.*, 2004). A clear outlier is fruit set in *Schisandra sphenanthera*, whose primary pollinator is gall midges, and for which thrips contribute only 5 % to fruit set (Du *et al.*, 2012). Seed set in the orchid *Habenaria radiata*, morphologically adapted to pollination by hawkmoths, was also an outlier with around 20 % set caused by thrips alone (Shigeta and Suetsugu, 2020). Several of the species are either dioecious or have self-incompatible bisexual flowers. Examples of dioecious species include *Macaranga hullettii*, *Castilla elastica* (androdioecious), *Antiaropsis decipiens* and *Schisandra sphenanthera*, and both *Arctostaphylos pungens* and *Stellera chamaejasme* are self-incompatible. All of these except *Schisandra* had around 50 % or higher fruit or seed set, and results are not owing to self-pollination. Therefore, the thrips-only treatments clearly demonstrate that thrips move between flowers, carry pollen and cross-pollinate.

There are several reasons as to why our quantitative analysis may be an underestimate of the contribution of thrips to pollination both in these experiments as well as in nature. First, it is important to note that the experimental studies included in our analyses are not an objective representation of insect-pollinated plants globally. These experiments focused on plants for which thrips pollination was already suspected either as co-pollinator or primary pollinator, and they met all the criteria required for the analyses. There are additional studies that (quantitatively) revealed the importance of thrips as pollinators but were not included in our analyses because not all required information was available. For example, in *Macrozamia lucida*, *Cycadothrips* is the sole pollinator found in cones of this cycad, and experiments showed that only ~100 thrips moving into ovulate cones were needed to equal the open pollination levels (Terry *et al.*, 2005), where more than a thousand thrips were captured landing on ovulate cones. Second, the thrips-only treatment group quantifies the relative importance of thrips to plant reproductive success in the absence of other insects. We do not know if thrips’ behaviour and dynamics in flowers are significantly different when other insects are barred, though we have no reason to expect any difference. Third, pollination levels under the treatment with coarse mesh size might (i.e. the thrips-only treatment) reduce pollination by thrips because they are physically hindered by or lose pollen on the mesh bags (Scott-Brown *et al.*, 2019). For example, the number of thrips per flower on *Stellera chamaejasme* was found to be only ~60 % when covered with coarse-meshed bags as compared with the number in fully open flowers (Zhang *et al.*, 2021), and seed and fruit set was lower in the thrips-only treatment (Fig. 3). Fourth, flower-bagging experiments designed to test for the importance of insect versus wind pollination commonly use a coarse mesh size (~0.5–1 mm) to exclude pollinators, but that mesh size does not prevent thrips from entering. In such cases, seed/fruit set may be attributed to wind pollination albeit it was (partly) caused by thrips. Finally, we can conclude that although few studies have quantified the importance of thrips in plant reproduction, it is clear that thrips significantly contribute to fruit and seed set.

## IS THERE A ‘THRIPOPHILY’ POLLINATION SYNDROME?

Despite their diminutive size, flower-visiting thrips appear no different in how they locate flowers compared with other floral insect visitors. Thrips respond to olfactory and vision stimuli (reviewed by Kirk *et al.*, 2021; Lopez-Reyes *et al.*, 2022). In addition, other cues, such as heat, CO_2_ and humidity, may play a role in attracting thrips (Salzman *et al.*, 2023; Terry, 2023), although understanding the relative importance of these cues would benefit from further study (van der Kooi *et al.*, 2023). Thrips species range from being highly polyphagous to highly specialized (reviewed by Mound, 2004), and with thrips from many genera being floral visitors, the question of how they find flowers may depend upon different plant traits. For example, particular colours can be sufficient to attract the western flower thrips, *Frankliniella occidentalis* (Matteson and Terry, 1992), and yellow tea thrips, *Scirtothrips dorsalis* (Kishi *et al.*, 2014), though adding plant chemical compounds often enhances the behavioural response (Mainali and Lim, 2011; Kirk *et al.*, 2021). Kirk (1985) found that pan traps painted white and laced with the commonly found floral volatile anisaldehyde caught many times more of six generalist flower-feeding thripid species than unlaced control traps with the same colour. By contrast, *Cycadothrips* species are highly specific to their host *Macrozamia* cycad species, and particular host cone volatile compounds alone attract their thrips pollinator (Terry *et al.*, 2021).

Pollination syndromes have been proposed to group the traits of plants, or the congruence of traits across a diversity of plants, that is associated with pollination by a particular pollinator guild (Faegri and Van der Pijl, 1979; Ollerton *et al.*, 2009). Recognizing that there are limitations with the pollination syndrome concept (Fenster *et al.*, 2004; Ollerton *et al.*, 2009; Ohashi *et al.*, 2021; Assis, 2023), characterizing a common suite of floral traits can be a starting point for analysing plants in a functional context. A thrips pollination syndrome, ‘thripophily’, has been proposed as a combination of the following floral traits: medium-sized flowers, that are ‘white to yellow, sweetly scented, with or without nectar, with compact floral structures, globose or urn-shaped blossoms providing shelter, and with small- to medium-sized pollen grains, possibly with nocturnal pollen presentation’ (Kirk, 1988). Given that only a few tropical plants have been examined (Kirk, 1997) and that there have been many recent studies of pollination by thrips, we here discuss broader indications of a thrips pollination syndrome.

### Visual display

Inflorescences visited by pollinating thrips (e.g. Figs 1 and 2) vary in size and colour. Many of the flowers or inflorescences listed for both *Frankliniella tritici* and *F. schultzei* are small (a few millimetres in diameter) to medium (a few centimetres) in size and colours range from white (e.g. *Prunus laurocerasus*), pale yellow to blue, pink and orange (e.g. California poppy, *Eschscholzia californica*). Gottsberger (1999) suggested that thrips-pollinated Annonaceae flowers are typically whitish. In contrast, the pollen and ovulate cones of *Cycadothrips*-pollinated *Macrozamia* cycads can be large, 20 cm in height or more, and are green to yellow. Individual sporophylls within cones are small- to medium-sized, up to ~2–3 cm across. When we look more broadly at plants likely pollinated by thrips from our literature search (Supplementary Data), it is clear that the size and colour of the dominant visual display varies across plant species. White and yellow flowers are common for thrips pollination, but these colours are also among the most common floral colours (Chittka *et al.*, 1994) and these colours do not exclude other insects, nor do generalist thrips species visit white and yellow flowers only. Crucially, the spectral characteristics of thrips-pollinated flowers, as well as thrips responses to these features, are virtually uncharted. It is thus impossible to quantify chromatic and achromatic contrast values for thrips-pollinated flowers, which are two key predictors for visibility of flowers to insects (Kemp *et al.*, 2015; van der Kooi and Kelber, 2022; van der Kooi and Spaethe, 2025).

### Volatile organic compounds

Very few studies have examined the odour characteristics of reproductive organs of thrips-pollinated plants. The best-studied attractants of thrips have been for pestiferous thrips because they are important in protecting crops and are an aid in monitoring pests. A review of semiochemicals produced by plants that attract thrips mentions that many flowers, but not all, have a fruity or sweet odour (Kirk *et al.*, 2021). For example, the strong bitter almond-smelling salicylaldehyde attracts *Frankliniella occidentalis* and *F. tritici*, and *Haplothrips aculeatus* is attracted by cheesy-scented compounds (Holtmann, 1963; Kirk *et al.*, 2021). The hops-like odour of β-myrcene monoterpene is associated with cones of some thrips-pollinated *Macrozamia* species, but other species cones have waxy fatty acid or floral odour (Terry *et al.*, 2021). For other thrips pollination studies, only a few even mention the organoleptic properties of the flower and even fewer have tested floral odours for attraction. Sweet-scented flowers are noted on *Pandanus odorifer* associated with *Amystrops* sap beetles and *Projectothrips* sp. and in the *Chamaedorea* palms associated with *Brooksithrips chamaedoreae* (Henderson, 2024; Miyamoto *et al*., 2024). Descriptions of different species’ odours within thrips-pollinated Anonnaceae range from rancid–fruity to sweet and perfumed (Gottsberger, 1999).

Only a few thrips-pollinated plants have been examined for particular floral volatile organic compounds (VOCs), by gas chromatographic and mass spectrographic techniques (GC–MS), to identify and quantify the profiles of floral odour components. Even fewer species and compounds have been tested for attraction. Pellmyr *et al.* (1990), studying flowers of Winteraceae in New Caledonia, found that the thrips *Taeniothrips novocaledonensis* is abundant on the flowers of three species of *Belliolum*. The primary *Belliolum* floral volatiles (only one plant flower was sampled) are the monoterpenoids linalool and linalool oxide. Other Winteraceae have different floral VOCs and are associated with other insects. Scott-Brown *et al.* (2019) demonstrated that the polyphagous *Thrips major* is the pollinator of *Sambucus nigra*. The major floral volatile component linalool is highest during the most receptive stage of floral development and during the time of day when thrips are most attracted to flowers, although other components are also present. They further demonstrated that two cyanogenic glycosides extracted from floral and surrounding tissues are highest at both early and post-floral stages, and these compounds deter thrips. The combination of attractive and deterrent compounds likely affects thrips abundance in these flowers. For these two studies on *Belliolum* and *Sambucus*, the common dominant floral volatile is linalool, a common monoterpene among flowers (Raguso and Pichersky, 1999), which raises questions about the thrips attraction. Why do these flowers attract only thrips and not other pollinators, and are there other attractants and/or repellents in the floral bouquet or floral morphology that account for the thrips attraction?

In Australia, many *Macrozamia* cycads have been examined for their cone VOCs. The first studies demonstrated a stark difference between a *Tranes* weevil-pollinated species and a *Cycadothrips chadwicki*-pollinated one (Terry *et al*., 2004) in southeast Queensland. The weevil-pollinated *M. machinii* cone odour is dominated by linalool and has other minor levels of monoterpenes, whereas cones of the thrips-pollinated *M. lucida* have no detectable linalool, but they have 100 times or more β-myrcene and other monoterpenes (Terry *et al.*, 2007). Later studies demonstrated that some *C. chadwicki*-pollinated *Macrozamia* species attract thrips with unique esters or fatty acid hydrocarbons (Terry *et al.*, 2021) and not with monoterpenes. Molecular studies of *C. chadwicki* across different species of *Macrozamia* in eastern Australia revealed that there are at least five different cryptic thrips species, separating mainly by geography (Brookes *et al.*, 2015). Intriguingly, host species’ specific cone odours are associated with this geography, and thrips are attracted to only their own host’s volatile compounds (Terry *et al.*, 2021; I. Terry et al., unpubl. res.). This suggests that thrips and their *Macrozamia* hosts have co-diversified and these changes have occurred over only a few million years (Brookes *et al.*, 2015).

### Pollen size and morphology

**Fig. 4.**
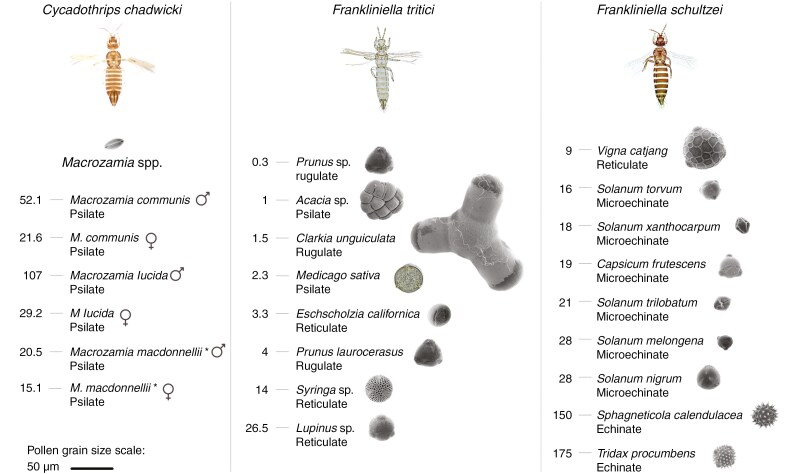
Visual summary of pollen carrying in three well-studied pollinating thrips species. The number of average pollen grains per individual is given per plant species. If known, pollen size and surface structure are shown on the right. For *Macrozamia*, pollen images of individual species are not shown, but pollen morphology of most cycad species is similar in size and structure (psilate; Dehgan and Dehgan, 1988). Pollen images are from paldat.org, except for *Solanum* (Vijayakumari and Vilasini, 2005; Ashfaq *et al.*, 2020). *Note that *Cycadothrips chadwicki* pollinates *Macrozamia communis* and *M. lucida*, but *C. albrechti* pollinates *M. macdonnellii*.

Thrips’ successful acquisition of nutrition from pollen grains, or any other plant part, is achieved via a ‘punch and suck’ feeding mechanism. The pollen grain wall is pierced with the protractable needle-like left mandible when punching against the grain, and contents are sucked through the food canal formed by maxillary stylets that insert at the puncture area (Kirk, 1984). Florivorous thrips are thus well adapted for obtaining nutrients from the insides of pollen grains, even very small grains. Although thrips can feed on pollen grains of various sizes and shapes, it stands to reason that their minute size might hinder adhesion of pollen to thrips bodies. If this is the case, we may expect that plants that are primarily pollinated by small insects have evolved pollen morphologies that enhance pollen adhesion, such as small sizes and spiny exine coatings (Tanaka *et al.*, 2004). For three thrips species with sufficient data available we explored whether there are commonalities in pollen size and shape among the plants that they pollinate, and whether there is an association between pollen morphology and thrips’ pollen load (Fig. 4). It seems that grains on average are on the smaller size; however, this is also a common phenomenon for other pollinators, including some larger beetles and bees (Hao *et al.*, 2020). For *Frankliniella schultzei*, echinate (spiny) pollen of two species is carried in more than five times higher numbers than pollen without spines, supporting the idea that spininess enhances pollen adhesion (Fig. 4), although this could also be caused by the number of available pollen grains of different plant species. Contrary to the general hypothesis that zoophilous pollen tends to be sticky and wind-vectored pollen is dry and powdery, pollen of several thrips-pollinated species is powdery, dry and forms smaller clumps. For example, the main pollinators of the orchid *Epipactis thunbergii* are hawkmoths, but thrips are found to also carry abundant granular pollen that separates from the pollinia (Suetsugu *et al.*, 2019).

### Floral structures that provide shelter or refuge

Thrips are well known for moving into tight places (Lewis, 1973), so any flower or cone with small or slit-like openings or a tubular shape may be favoured by thrips. Globose flowers, such as clover, disc flowers of asters and urecolate (urn-like) flowers such as those of Ericaceae have tight places or entries where thrips and other small insects can enter. Some of these have small ostioles or slits for entry to reproductive organs that do appear to exclude other larger pollinators, e.g. *Wilkiea hugeliana* (Williams *et al.*, 2001) and *Castilla elastica* (Sakai, 2001). Whereas some of these floral types may not exclude all other pollinators, there are numerous examples of reproductive parts with closed or tight structures where thrips are pollinators and sometimes the sole pollinator. Some examples include *Cycadothrips* on *Macrozamia* cycads; *Thrips antiaropsidis* on *Antiaropsis decipiens*; *Brooksithrips* on *Chamaeodorea* palms; *Projectothrips* on *Pandanus odorifer*; and *Dolichothrips* on *Macaranga* (Mound and Terry, 2001; Moog *et al.*, 2002; Zerega *et al.*, 2004; Morgan, 2007; Fiala *et al.*, 2011; Ríos *et al.*, 2014). In *Macrozamia* ovulate cones, the sporophylls are packed together, and the micropylar tip is inwardly facing so that only small and dorsoventrally flattened insects can navigate through the tight opening to deliver the pollen to the micropyle.

### Thermogenesis

Some plants’ reproductive organs can increase their temperature relative to the ambient conditions by ramping up their respiratory metabolism (Seymour and Schultze-Motel, 1996; Thien *et al.*, 2000; van der Kooi *et al.*, 2019; Claudel *et al.*, 2023). Thermogenesis has been proposed as an ancient trait involved in early pollination (Peris *et al.*, 2024); however, most of the thrips-pollinated plants are not known to be thermogenic. Some palm species’ flowers, for example, are thermogenic, but there was either no evidence of, or no attempted measurement of, thermogenesis in thrips-pollinated *Chamaedorea* (Henderson, 2024). Some *Pandanus* species are thermogenic, including *Pandanus odorifer* (Miyamoto *et al.*, 2024), which is pollinated mainly by *Amystrops* sap beetle and secondarily by *Projectothrips* sp. (Mound and Tree, 2011). Miyamoto *et al.* (2024) mentioned that thermogenesis occurred at night when the sap beetles were actively visiting flowers, but specific thermogenic effects on both these pollinators were not reported.

One group of plants well known for their thermogenesis is the cycads, and in most species both pollen and ovulate cones have a daily thermogenic event for many days (Tang, 1987). In *Macrozamia* the thermogenic signals are associated with the daily routine of the *Cycadothrips* pollinator behaviour (Terry *et al.*, 2004, 2014; Terry, 2023). Many thrips-pollinated species are thermogenic for several hours during the mid-day period, while *Tranes* weevil-pollinated *Macrozamia* are heating around sunset and evening (Terry *et al.*, 2004). During these events, the respiratory metabolism ramps up (Roemer *et al.*, 2012; Terry *et al.*, 2016) causing an increase in cone temperature, and then volatile production and emission increase. Throngs of adult thrips exit cones en masse during the peak of each thermogenic event (Fig. 2C). As respiration rate slows down, temperature and volatile emissions decrease, and thrips become attracted to cones later in the day. In these cycads, warm cone temperatures, volatiles and humidity, as by-products of the high respiration, mediate thrips behaviour, and this system has been characterized as a multimodal signalling ‘push–pull’ pollination (Terry *et al.*, 2007). This indicates how complex and intricate this mutually obligate pollination system may be. Whether any of the other tight pollination mutualisms involve thrips and thermogenic flowers needs further investigation.

## CONCLUDING REMARKS

Darwin, (1876), being the consummate observer, noticed small pollen-carrying insects, specifically thrips, that could not be barred from flowers easily. He wondered if they might cross-pollinate the plants he had intended for self-fertilization. Indeed, we can conclude that thrips are more important as pollinators than commonly considered. Our survey has identified that thrips are pollinators across the globe and across gymnosperms and angiosperms (Fig. 1). Many thrips pollination systems are more generalized (Kondo *et al.*, 2016; Suetsugu *et al.*, 2019; Shigeta and Suetsugu, 2020), sometimes in combination with wind pollination (Ríos *et al.*, 2014). Some pollinating thrips visit flowers of various species (*Frankliniella*, Fig. 1). However, we are finding that many plant species are specialized for thrips pollination, such as in Cycadales, Castilleae, palms and *Macaranga* (Mound and Terry, 2001; Sakai, 2001; Moog *et al.*, 2002; Zerega *et al.*, 2004). Our meta-analyses revealed that, on average, thrips contribute 55 % of seed set and 38 % of fruit set, although even values close to 90 % occur (Zerega *et al.*, 2004; Eliyahu *et al.*, 2015). Additional evidence to support the importance of thrips as pollinators comes from dioecious and self-incompatible species in which reproductive organs are only accessed by thrips. There is no set of floral traits that define thrips pollination, presumably because of the diversity of flower-visiting thrips and their preferences. For some host-specific thrips, a specific floral architecture, along with visual and/or olfactory cues, is critical for attraction, although the sensory systems of thrips and their behavioural responses are only beginning to be understood (Lopez-Reyes *et al.*, 2022).

Finally, we propose several open questions that will help us propel forward our understanding of plant and insect co-evolution:

(1) Is there parallel or convergent evolution of floral traits in unrelated thrips-pollinated taxa?
(2) What is the pollination efficiency of thrips, and how is that mediated by floral or pollen traits, and thrips dispersal?
(3) What are the nutritional preferences of thrips, and how have they shaped the pollen chemical profiles of plants that are pollinated by thrips (e.g. Ruedenauer *et al.*, 2019)?
(4) How do specialist thrips choose their hosts, and what floral cues are important?
(5) If thrips pollination occurs in conjunction with flower visitation/pollination by other animals, when are thrips mutualists and when are they antagonists (Thomson, 2003; van der Kooi *et al.*, 2021)?

Understanding these aspects is not only of fundamental interest, but also bears applied relevance, e.g. for the design of new biological controls, which is warranted given the enormous damage to our environment caused by insecticides. We encourage the flower and pollination community to consider thrips as (potential) pollinators and study systems, because they are tiny but can be significant!

## Supplementary Material

mcaf069_suppl_Supplementary_Data
